# Effectiveness of bariatric surgery on body mass, biochemical parameters, and steatosis in metabolically healthy vs. unhealthy obesity

**DOI:** 10.1590/0102-67202025000030e1899

**Published:** 2025-10-10

**Authors:** Ana Paula de Sousa ITO, Lindsey Mikulski ITAHIDES, Rosane Aparecida RIBEIRO, Maria Lúcia BONFLEUR

**Affiliations:** 1 Universidade Estadual do Oeste do Paraná, Centro de Ciências Biológicas e da Saúde – Cascavel (PR), Brazil.; 2 Universidade Estadual de Ponta Grossa, Setor de Ciências Biológicas e da Saúde, General Biology – Ponta Grossa (PR), Brazil.

**Keywords:** Hepatopatia gordurosa não alcoólica, Obesidade, Derivação gástrica, Gastrectomia, Non-alcoholic fatty liver disease, Obesity, Gastric bypass, Gastrectomy

## Abstract

**Background:**

The effects of bariatric surgery in metabolically healthy obese (MHO) versus metabolically unhealthy obese (MUO) patients are underexplored in the literature.

**Aims:**

The aim of the study was to compare the impact of bariatric surgery on weight loss, body composition, plasma biochemical parameters, and hepatic steatosis in MHO and MUO individuals.

**Methods:**

Preoperative and 1-year postoperative medical records of 82 men and women aged 18–65 years, with body mass index >30 kg/m_2_, who underwent bariatric surgery from September 2021 to March 2023 were analyzed. MUO individuals were defined as those, metabolically unhealthy obese, with two metabolic syndrome risk factors, in preoperative data.

**Results:**

The prevalence of MHO and MUO individuals was 22 and 78%, respectively. Preoperative neck circumference and visceral adiposity index were higher in MUO individuals. Hepatic steatosis was the most common comorbidity in both groups. After 1 year, both groups demonstrated similar benefits from bariatric surgery in reducing body weight, adiposity, and anthropometric indices. Bariatric surgery also improved blood glucose, insulin sensitivity, and dyslipidemia in MUO individuals. However, 30% of MUO individuals presented with steatosis, compared to only 5.6% of MHO individuals. This outcome was accompanied by higher plasma levels of ferritin, alanine aminotransferase, and aspartate aminotransferase in MUO individuals.

**Conclusions:**

Bariatric surgery provided similar benefits in body mass for MHO and MUO individuals. However, after 1 year, MUO individuals still exhibited elevated markers of inflammation, liver injury, and steatosis, suggesting greater residual metabolic vulnerability.

## ARTICLE HIGHLIGHTS

Bariatric surgery improved biometric parameters similarly in both MUO and MHO individuals.

Hepatic steatosis was reduced in both MUO and MHO groups following bariatric surgery.

Approximately 30% of MUO subjects continued to exhibit steatosis and elevated markers of hepatic inflammation and injury after surgery.

## Summary

**CENTRAL MESSAGE:**

Obesity is a multifaceted and pervasive public health challenge related to comorbidities such as type 2 diabetes mellitus, cardiovascular disease, sleep apnea, kidney disease, some types of cancer, and non-alcoholic fatty liver disease. It is reported that around 15%–36% of people with obesity display preserved metabolic health and do not exhibit such obesity comorbidities. These subjects are called metabolically healthy obese, in which the body fat excess is not accompanied by insulin resistance, dyslipidemia, or hypertension. On the other hand, most obese individuals present impairments in metabolic health and the comorbidities of obesity; thus, these are referred to as metabolically unhealthy obese.

**PERSPECTIVES:**

Bariatric surgery led to a similar effectiveness in weight loss, reductions in adiposity and anthropometric indices, and improvement in plasma glucose, lipids, insulin sensitivity, and renal parameters among metabolically unhealthy obese (MUO) and metabolically healthy obese (MHO) individuals. However, the effects of bariatric surgery between the MUO and MHO phenotypes differed significantly on hepatic steatosis and biochemical parameters of inflammation and liver damage, indicating that 1 year of the surgical intervention has better outcomes on hepatic steatosis in MHO individuals.

## VISUAL ABSTRACT

**Figure:**
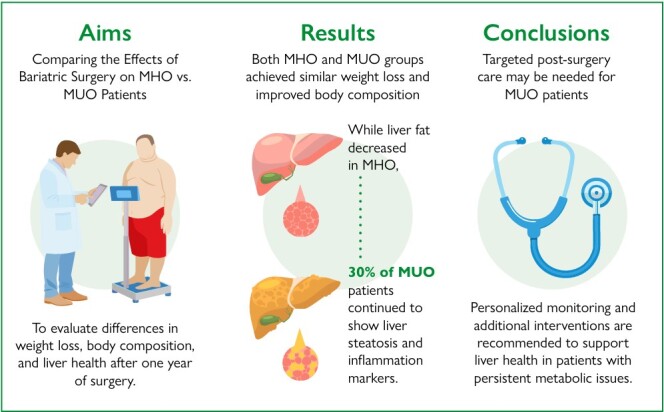

## RESUMO

**Racional:**

O efeito da cirurgia bariátrica em pacientes metabolicamente saudáveis (MHO) vs. não saudáveis (MUO) é pouco explorada na literatura.

**Objetivos:**

Comparar os efeitos da cirurgia bariátrica na perda de peso, composição corporal, parâmetros bioquímicos plasmáticos e esteatose hepática em indivíduos MHO e MUO.

**Métodos:**

Foram analisados os registros médicos pré-operatórios e pós-operatórios de um ano de 82 homens e mulheres, com idades entre 18 e 65 anos, com IMC > 30 kg/m^2^, que foram submetidos à cirurgia bariátrica entre setembro de 2021 e março de 2023. Indivíduos MUO foram definidos como aqueles que apresentavam dois fatores de risco para síndrome metabólica nos dados pré-operatórios.

**Resultados:**

A prevalência de indivíduos MHO e MUO foi de 22 e 78%. A circunferência do pescoço e o índice de adiposidade visceral pré-operatórios foram maiores nos indivíduos MUO. Esteatose hepática foi a comorbidade mais comum nos dois grupos. Após um ano, ambos os grupos apresentaram benefícios semelhantes da cirurgia bariátrica na redução de peso corporal, adiposidade e índices antropométricos. A cirurgia bariátrica também melhorou a glicemia, a sensibilidade à insulina e a dislipidemia nos indivíduos MUO. No entanto, 30% dos indivíduos MUO apresentaram esteatose, enquanto apenas 5,6% dos MHO exibiram esteatose. Esse efeito foi acompanhado por níveis plasmáticos mais altos de ferritina, ALT e AST nos indivíduos MUO.

**Conclusões:**

A cirurgia bariátrica promoveu benefícios similares na massa corporal entre MHO e MUO, mas os indivíduos MUO mantiveram, após um ano, marcadores elevados de inflamação, lesão hepática e esteatose, sugerindo maior vulnerabilidade metabólica residual.

## INTRODUCTION

 Obesity is a multifaceted and pervasive public health challenge responsible for the loss of life years due to this disease and its related comorbidities such as type 2 diabetes mellitus, cardiovascular disease, sleep apnea, kidney disease, some types of cancers^10^, and non-alcoholic fatty liver disease (NAFLD)^15 ,29^. Notably, it is reported that around 15–36% of people with obesity display preserved metabolic health and do not exhibit such obesity comorbidities^5,6,11,21,22 ,24,26 ,29^. These subjects are called metabolically healthy obese (MHO), in which the body fat excess is not accompanied by insulin resistance, dyslipidemia, or hypertension. On the other hand, most obese individuals present impairments in metabolic health and the comorbidities of obesity; thus, these are referred to as metabolically unhealthy obese (MUO)^18^. 

 Bariatric surgery has emerged as an important therapeutic option for obesity and its comorbidities. The most performed surgical techniques worldwide are vertical sleeve gastrectomy (SG) and Roux-en-Y gastric bypass (RYGB)^7^. These surgical procedures facilitate weight loss and improve insulin sensitivity, body glucose control, dyslipidemias, and hypertension^16^. Interestingly, the degree of such bariatric-related effects may vary among population with obesity, and studies suggest that MHO individuals can benefit from bariatric surgery in terms of metabolic improvements, even if they do not have many of the pre-existing metabolic abnormalities as MUO individuals^11 ,26^. Therefore, there are few studies in the literature comparing the effects of bariatric surgery on MHO and MUO phenotypes, particularly regarding its therapeutic actions against hepatic steatosis, the first stage of NAFLD, a condition often associated with metabolic syndrome^14^, but that also affects MHO subjects^9^, and currently represents the main liver disease worldwide^17 ,30^. Therefore, the objectives of this research are to analyze the effectiveness of bariatric surgery between MHO and MUO subjects, focusing on differences in body mass index (BMI), biochemical parameters, and hepatic steatosis after 1 year of surgery. 

## METHODS

### Study design and population

 This is a quantitative study of retrospective longitudinal documentary analysis, performed from September 2021 to March 2023, in a specialized gastroenterology service in Cascavel, PR, Brazil. The clinic holds the ONA (*Organização Nacional de Acreditação* – National Accreditation Organization) level 3 accreditation certificate, which ensures healthcare services meet quality and safety standards. This study was approved by the Research Ethics Committee at Universidade Estadual do Oeste do Paraná (UNIOESTE) (Protocol n.: 5.670.236) and by the Research Ethics Committee of the Universidade Estadual de Ponta Grossa (UEPG), Cascavel, (PR). 

 Data collection comprised a total of 102 eligible participants, who were both men and women, aged 18–65 years, exhibiting a BMI >30 kg/m^2^, and who underwent RYGB or SG surgery. Twenty subjects were excluded: 13 patients without 1-year post-surgery follow-up, two patients who underwent revisional bariatric surgeries, and five pregnant women, totalizing 82 subjects in the casuistic analyzed. 

### Definition of metabolically healthy obese and metabolically unhealthy obese phenotypes

 The MHO and MUO phenotype classification was based on the previous metabolic syndrome risk factors study reported by Alberti et al.^2^. Thus, metabolic syndrome was considered when patients displayed at least three of the risk factors: elevated blood pressure, with systolic (SBP)/diastolic blood pressure (DBP)=130/85 mmHg, or if they use antihypertensive medication; increased triglycerides (TG) plasma levels: =150 mg/dL, or if they use hypolipidemic medications; reduced high-density lipoprotein (HDL) plasma levels: =40 mg/dL for men and =50 mg/dL for women, or if they use hypolipidemic medications; and increased fasting glycemia: =100 mg/dL, or if they use hypoglycemic medication. Therefore, the MHO group included patients that had BMI=30 and showed two or more metabolic syndrome risk factors; whereas the MUO group included patients with BMI=30 and who had three or more metabolic syndrome risk factors. 

### Data collection

 Data from medical records of the 82 subjects of the preoperative period and after 1 year of surgical procedures were obtained through the Medical Services Management System, Sisclínica^®^ (Florianópolis, SC, Brazil), from September 2021 to March 2023. The general variables collected were as follows: gender, age, body weight (BW), height, BMI, surgical technique performed (RYGB or SG), neck circumference (NC), waist circumference (WC), hip circumference (HC), SBP and DBP, use of antihypertensive and hypoglycemic medications, and presence and grade of hepatic steatosis. 

 Fatty liver was detected using the Toshiba Model Aplio 300 ultrasound system (Toshiba Medical System, Tokyo, Japan). This bidimensional model employs a dynamic, sectoral, and linear equipment operating at frequencies between 3.5 and 6 MHz. The criteria for steatosis quantification were based on the classification developed by Saadeh et al.^27^. 

 The anthropometric parameters obtained were percentage of total body fat, muscle mass (MM), fat-free mass (FFM), and basal metabolic rate (BMR). These parameters were obtained through bioimpedance using the InBody^®^ Model S10 equipment (InBody Co., Seoul, South Korea). 

 The plasma variables obtained in medical records included fasting glycemia, insulinemia, total cholesterol (CHOL), high-density lipoprotein (HDL), very low-density lipoprotein (VLDL), low-density lipoprotein (LDL), TG, aspartate aminotransferase (AST), alanine aminotransferase (ALT), gamma-glutamyl transferase (GGT), ferritin, creatinine, and urea. The homeostasis model assessment of insulin resistance index (HOMA-IR) was calculated by multiplying fasting glycemia (mmol/L) by fasting insulinemia (μU/mL) and dividing by 22.5^20^. The visceral adiposity index (VAI) was calculated, as previously described by Amato et al.,^3^ using the formula for men: [((WC/39.68+(1.88xBMI))xTG/1.03x1.31/HDL)]; and for women: [((WC/36.58+(1.89xBMI))xTG/0.81x1.52/HDL)]. 

### Statistical analysis

 Qualitative data were presented as absolute or relative frequencies and assessed using the chi-square test for independence. When theoretical counts were less than 5, the Monte Carlo’s method was applied. Quantitative data were presented as means±standard deviation (SD). First, data were analyzed using the Shapiro-Wilk test for verification of normality and homoscedasticity. Subsequently, the data were compared using unpaired and paired parametric (Student’s t-test) or nonparametric (Mann-Whitney test and Wilcoxon test) tests. The levels of significance were set at p<0.05. Delta values were obtained by subtracting postoperative values from preoperative values and were presented as means±SD. 

 Principal component analysis (PCA) was conducted, involving a matrix with the variables: BMI, NC, WC, HC, glucose, insulin, LDL, TG, and HDL plasma levels. The KaiserMeyer-Olkin (KMO) test was applied, and when KMO was >0.5, it was considered adequate for analysis. The PCA defined the factorial loads, representing linear correlations (Pearson) of each variable with the factorial composition. These factorial loads define the factors, new variables derived from the set of factorial loads. To assess the cumulative percentage variation, eigenvalues were calculated, identifying the contributions of individual dimensions and the influence of relevant variables. Using the "ggplot2" package, a diagram was constructed to arrange the PCA eigenvectors, employing ellipses to visually separate the MHO (pre and post) and MUO (pre and post) groups, facilitating the identification of linear associations between variables and groups in the first two dimensions. 

 Statistical analyses for quantitative data were performed using GraphPad Prism 10 version 10.0.1 (GraphPad by Dotmatics, Boston, MA, USA), while qualitative data and PCAs were executed in R software, version 4.3.2 ( https://www.rproject.org/about.html).

## RESULTS

 Eighty-two patients were included in the present study, and their general characteristics are presented in Table 1. Most of the data obtained in this retrospective longitudinal documentary analysis were from women, and the BMI of the eligible participants was 40.13±4.60, with the majority classified with grade 3 obesity. The most performed surgical procedure was RYGB (78%), and hepatic steatosis was present in 91.5% of the patients. Thirty-one-point-seven percent (31.7%) of the patients displayed hypertension (Table 1). 

**Table 1:** General characteristics of all eligible participants and in the metabolically healthy obese and metabolically unhealthy obese phenotypes observed before the bariatric procedure.

		Total n=82 (100%)	MHO n=18 (22%)	MUO n=64 (78%)	p-value MHO×MUO Gender n (%)
Gender					
	Women	69 (84.10)	16 (88.90)	53 (82.80)	0.5329*
	Men	13 (15.90)	2 (11.10)	11 (17.20)	
Age (years)		37.62±10.60	32.00±6.70	39.00±11.00	0.0195†
High (m)		1.65±0.08	1.66±0.07	1.64±0.08	0.4692‡
Weight (kg)		109.5±18.5	110.0±18.0	109.0±19.0	0.9108†
BMI (kg/m^2^)		40.13±4.60	39.90±4.90	40.20±4.50	0.7613†
Obesity grade n (%)					
	Grade I	5 (6.10)	2 (11.20)	3 (4.70)	0.7022‡
	Grade II	35 (42.70)	8 (44.40)	29 (45.30)	
	Grade III	42 (51.20)	8 (44.40)	32 (50.00)	
Type of surgery , n (%)					
	RYGB	64 (78.00)	14 (77.80)	50 (78.10)	0.9749*
	SG	18 (22.00)	4 (22.20)	14 (21.90)	
Hepatic steatosis					
	Present, n (%)	75 (91.50)	16 (88.90)	59 (92.20)	0.6581*
	Absent, n (%)	7 (8.50)	2 (11.11)	5 (7.81)	
Hypertension n (%)					
	Present	26 (31.70)	0 (0.00)	26 (40.62)	0.0011*
	Absent	56 (68.30)	18 (100.00)	38 (59.37)	0.1143*
SBP (mmHg)		127±9.40	123±7.80	130±11.00	0.0235†
DBP (mmHg)		81±5.90	80±4.00	82±6.30	0.4363†

The data are presented as n (%) or means±SD. SD: standard deviation.

* ꓓ^2^ test for Independence

† Mann-Whitney test

‡ Student’s t-test. Wilcoxon-Mann-Whitney test.

MHO: metabolically healthy obese; MUO: metabolically unhealthy obese; BMI: body mass index; RYGB: Roux-en-Y gastric bypass; SG: sleeve gastrectomy; SBP: systolic blood pressure; DBP: diastolic blood pressure.

 Using the criteria proposed by the Joint Scientific Statement for Defining Phenotypes^18^, before the bariatric surgeries, 22% (n=18) of the subjects were characterized as MHO and 78% (n=64) as MUO (Table 1). The age and SBP were 21.9% and 5.6% higher, respectively, in the MUO group when compared to the MHO. Additionally, 40.6% of the MUO group had hypertension. The steatosis scoring and other biometric parameters did not differ between the two phenotypes (Table 1). 

Table 2 shows that in the preoperative period, MUO subjects exhibited higher NC and VAI when compared to MHO subjects. The body mass, BMI, HC, WC, body fat, MM, FFM, and BMR did not differ between MUO and MHO groups in the preoperative period. One year after bariatric procedures, within the same phenotype analyzed, it was observed that the surgery efficiently reduced all biometric parameters available (Table 2). In addition, no differences in anthropometric and body mass composition parameters were observed between MUO and MHO subjects after one year of bariatric surgery. 

**Table 2:** Anthropometric characteristics and body composition in metabolically healthy obese (n=18) and metabolically unhealthy obese (n=64) phenotypes in pre- and postoperative periods.

		Groups	Preoperative period	p-value Preoperative MHO × MUO	Postoperative period	p-value Postoperative MHO × MUO	Delta values	p-value Preoperative x Postoperative
Weight (kg)		MHO	110.10±18.20	0.9108*	71.70±13.80	0.7749†	-38.38±11.25	<0.0001†
		MUO	109.30±18.70		74.20±18.00		-35.09±9.20	<0.0001†
BMI (kg/m^2^)		MHO	39.90±4.90	0.7613*	26.00±4.20	0.2572†	-13.89±3.58	<0.0001†
		MUO	40.20±4.50		27.10±4.70		-13.10±3.23	<0.0001†
NC (cm)		MHO	38.80±2.00	0.0347†	33.20±3.00	0.0943†	-5.67±2.64	<0.0001†
		MUO	41.00±3.80		34.90±3.90		-6.07±2.17	<0.0001†
VWC (cm)		MHO	110.90±12.60	0.8133†	79.20±9.80	0.1233†	-31.69±11.57	<0.0001†
		MUO	111.00±12.80		84.50±13.60		-26.53±10.50	<0.0001*
RC (cm)		MHO	131.60±8.80	0.3375†	101.20±8.70	0.8046†	-30.44±9.22	<0.0001†
		MUO	129.20±9.70		102.50±10.60		-26.70±8.22	<0.0001†
VAI		MHO	2.90±0.90	0.0001*	2.00±0.80	0.0605†	-0.88±0.72	<0.0001†
		MUO	5.70±4.50		2.90±1.70		-2.71±3.96	<0.0001†
Body fat (%)		MHO	49.00±4.20	0.6387†	33.50±8.80	0.6644*	-15.48±6.27	<0.0001*
		MUO	47.80±5.40		32.50±8.80		-15.27±6.95	<0.0001†
MM (kg)		MHO	30.80±5.40	0.7202†	25.90±5.40	0.4749†	-4.92±2.86	<0.0001*
		MUO	31.80±7.50		27.30±7.20		-4.59±3.23	<0.0001†
FFM (kg)		MHO	55.90±9.70	0.9491†	47.40±8.80	0.4819†	-8.48±4.15	<0.0001*
		MUO	56.90±12.40		49.30±11.20		-7.55±4.70	<0.0001†
BMR (kcal)		MHO	1546.00±203.20	0.6112†	1394.00±192.70	0.4345†	-151.90±110.90	<0.0001*
		MUO	1593.00±284.80		1447.00±247.90		-145.50±97.29	<0.0001†

The data are means±SD. The Delta values are means±SD obtained by subtracting the postoperative values from the preoperative values.

* Student’s t-test

† Mann-Whitney test

SD: standard deviation; MHO: metabolically healthy obese; MUO: metabolically unhealthy obese; BMI: body mass index; NC: neck circumference; WC: waist circumference; RC: rip circumference; VAI: visceral adiposity index; MMM: muscle mass; FFM: fat-free mass; BMR: basal metabolic rate.

 Regarding plasma biochemical data in the preoperative period, MUO patients showed increased glycemia, insulinemia, HOMA-IR, VLDL, TG, ALT, GGT, ferritin, and urea, and reduced HDLc plasma levels, when compared to the MHO subjects (Table 3). After 1 year of bariatric surgery, MUO subjects displayed amelioration of various plasma biochemical parameters, showing reduced glycemia, insulinemia, HOMA-IR, total CHOL, LDLc, VLDL, AST, GGT, ferritin, creatinine, and urea plasma levels, while an increase in HDLc circulating levels was observed when compared with their data in the preoperative period (Table 3). In the MHO group, there were observed reductions in plasma total CHOL, LDL, VLDL, TG, and GGT concentrations after one year of the operation. However, when comparing MUO and MHO subjects, it was observed that MUO individuals, still after 1 year of bariatric operation, exhibited higher plasma levels of AST, ALT, and ferritin than those observed for MHO (Table 3). 

**Table 3:** Plasma biochemical parameters in metabolically healthy obese (n=18) and metabolically unhealthy obese (n=64) phenotypes in pre- and postoperative periods.

		Groups	Preoperative	p-value Preoperative MHO × MUO	Postoperative	p-value Postoperative MHO × MUO	Delta values	p-value Preoperative x Postoperative
Glucose (mg/dL)		MHO	85.7±6.2	<0.0001*	83.5±7.4	0.5799*	-2.23±8.68	0.2916†
		MUO	106.6±33.8		84.7±10.5		-21.9±28.27	<0.0001†
Insulin (U/mL)		MHO	11.6±8.3	0.0083†	6.3±4.2	0.3549*	-4.17±11.00	0.2734*
		MUO	21.5±13.0		7.2±4.4		-13.97±11.75	<0.0001*
HOMA-IR		MHO	2.4±1.9	0.0028*	1.3±0.9	0.3650*	-0.89±1.42	0.3396*
		MUO	5.7±4.3		1.5±1.0		-4.20±3.84	<0.0001*
CHOL (mg/dL)		MHO	189.5±35.1	0.9923*	160.6±20.0	0.5918*	-28.95±29.85	0.0008†
		MUO	189.6±32.3		159.8±30.2		-29.78±35.65	<0.0001*
LDLc (mg/dL)		MHO	109.0±28.2	0.6190*	87.0±27.3	0.5439^b^	-26.32±27.03	0.0007†
		MUO	111.3±31.9		82.7±24.0		-24.27±36.07	<0.0001*
HDLc (mg/dL)		MHO	60.1±13.2	<0.0001*	63.3±13.4	0.0891*	3.08±11.58	0.5014*
		MUO	45.4±11.0		56.5±13.7		11.11±12.21	<0.0001*
VLDL (mg/dL)		MHO	19.4±5.1	0.0034*	14.8±4.7	0.1294*	-4.64±5.85	<0.0001*
		MUO	28.4±14.0		17.8±7.7		-10.61±11.33	0.0039*
TG (mg/dL)		MHO	97.0±25.6	0.0034*	73.7±23.6	0.1128*	-23.21±29.23	0.0040*
		MUO	109.30±18.70		74.20±18.00		-35.09±9.20	<0.0001†
AST (U/L)		MHO	22.8±14.0	0.1026*	17.6±3.9	0.0077*	-5.23±11.99	0.1778*
		MUO	26.1±11.4		25.7±19.1		-0.36±22.97	0.0942*
ALT (U/L)		MHO	24.6±16.0	0.0451*	19.20±5.80	0.0174*	-5.39±13.84	0.3463*
		MUO	31.8±16.4		25.90±13.50		-5.92±20.10	0.0066*
GGT (U/L)		MHO	29.5±29.4	0.0036*	16.40±11.90	0.0642*	-13.08±20.10	0.0007*
		MUO	35.6±14.7		19.50±13.40		-16.16±16.26	<0.0001*
Ferritin (ng/mL)		MHO	67.2±61.3	0.0005*	68.40±79.90	0.0049*	-16.84±59.12	0.6441*
		MUO	172.9±172.0		140.80±122.20		-36.21±117.20	0.0066*
Creatinine (mg/dL)		MHO	0.78±0.14	0.6122†	0.76±0.14	0.8636*	-0.02±0.12	0.5499†
		MUO	0.80±0.20		0.76±0.15		-0.045±0.13	0.0066*
Urea (mg/dL)		MHO	25.2±9.2	0.0361*	26.7±8.1	0.2429*	1.39±12.88	0.4789*
		MUO	28.0±7.6		24.0±7.3		-4.02±8.38	0.0003*

The data are means±SD. The Delta values are means±SD obtained by subtracting the postoperative values from the preoperative values.

* Mann-Whitney test

† Student’s t-test

MHO: metabolically healthy obese; MUO: metabolically unhealthy obese; HOMA-IR: omeostasis model assessment of insulin resistance; CHOL: cholesterol; LDL: low-density lipoprotein; HDL: high-density lipoprotein; VLDL: very low-density lipoprotein; TG: triglycerides; AST: aspartate aminotransferase; ALT: alanine aminotransferase; GGT: gamma-glutamyl transferase.

**Figure 1:**
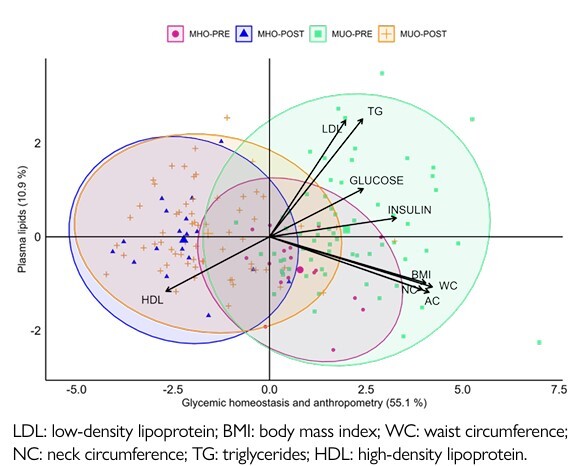
Principal component analysis (PCA). Ordination diagram of eigenvalues and eigenvectors, showing ellipses that separate the groups according to the periods pre and post-surgery.

 To explore the variation in MHO and MUO individuals before and after the bariatric procedure, PCA was utilized (Figure 1). The X-axis represents glycemic homeostasis and anthropometry, explaining 55.1% of the total data variation (eigenvalue=4.96). The Y-axis represents plasma lipids, explaining 10.9% of the total data variation (eigenvalue=0.98). Together, these two dimensions explain 66% of the total data variance. The ellipses indicate some overlap but also reveal clear distinctions between the pre- and post-intervention states in the MHO and MUO groups. MHO individuals exhibited less variation and more controlled changes postintervention, while MUO individuals showed greater dispersion in metabolic and anthropometric characteristics, indicating that bariatric surgeries may have differential effects on MHO and MUO individuals. 

**Figure 2:**
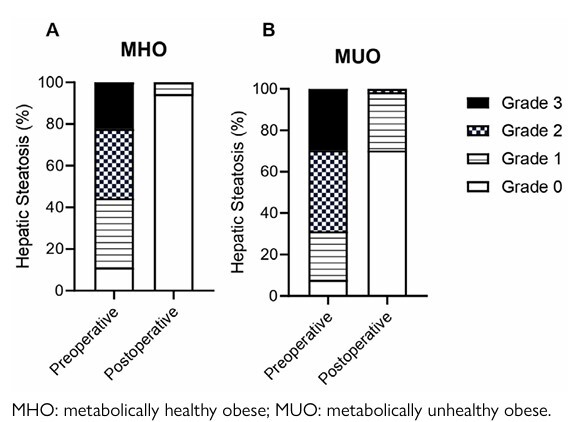
Hepatic steatosis grades. Histogram representing the percentage of hepatic steatosis grades in metabolically healthy obese (A) and metabolically unhealthy obese (B) subjects in pre- and postoperative period.

 Hepatic steatosis was an obesity comorbidity present in both MHO and MUO groups (Table 1), and as can be observed in Figure 2, prior to the bariatric intervention, 11.1% of MHO did not exhibit steatosis, 33.3, 33.3, and 22.3% displayed grades 1, 2, and 3 of steatosis, respectively (Figure 2A). In addition, only 7.8% of MUO subjects did not present hepatic steatosis, and 23.4%, 39.1%, and 29.7% exhibited grades 1, 2, and 3 steatosis (Figure 2B). Remarkably, 1-year post-bariatric procedure, 94.4% of MHO subjects did not show hepatic steatosis, and only 5.6% of MHO group displayed mild (grade 1) steatosis (Figure 2A). For the MUO group, bariatric surgeries also ameliorated hepatic steatosis, since 70.3% of MUO subjects did not display steatosis, 28.1% exhibited mild (grade 1) steatosis, and 1.6% showed moderate (grade 2) steatosis (Figure 2B). But is important to highlight that after 1 year of the surgical procedure, the percentage of hepatic steatosis in MUO groups, was statistically significantly higher than those observed for MHO (p<0.03, p>0.05). 

## DISCUSSION

 This study analyzed a series composed of 82 people with obesity, of whom 78% were classified as MUO and 22% were categorized as MHO. Such percentage is in accordance with the early reports of the prevalence of these obesity phenotypes, showing sample variation from 18 to 36% for the MHO subjects and from 64 to 82% for the MUO^5,6,11,21,22,24,26,29^. Also, here, the eligible participants are predominantly women, constituting 84.1% of the total sample, irrespective of the obesity phenotype. This women prevalence also was obtained by other studies, showing that women represent between 57 and 76% of the sample analyzed regarding to MH and MU obesity phenotypes^24,26^. In addition, as previously reported^5,22,24,26^ here the MHO group also consisted of younger subjects than those in the MUO. This higher incidence of younger subjects in the MHO group may be justified by the fact that this phenotype represents a transitional state toward the MUO phenotype, contingent upon the duration of exposure to obesity. The transition from the MHO to the MUO phenotype, when the disease is not controlled, seems to occur within a period of 7–8 years^1^. 

 The bioimpedance performed in the preoperative period demonstrated a similar fat distribution among MHO and MUO subjects (Table 2). However, NC and VAI in the MUO subjects were significantly higher than those in the MHO group. Although the NC values in both groups exceeded the cutoffs considered safe for cardiovascular health^23^, the NC in the MUO group was about 5.7% higher than in the MHO group, suggesting a higher risk of developing cardiovascular diseases and obstructive sleep apnea. The increased VAI in MUO subjects aids in differentiating between obesity phenotypes, being a useful tool for diagnosing body fat distribution, particularly subcutaneous fat, and its potential role in the pathogenesis of obesity-related comorbidities^3^. 

 Data of the postoperative period demonstrated that bariatric surgery led to a similar BW loss and reductions in adiposity and anthropometric indices in MUO and MHO subjects. These results agree with early studies that observed similar BW loss in MHO and MUO subjects after 1 year of bariatric surgery^12,19^, demonstrating that bariatric surgery has similar benefits on biometric parameters of MUO and MHO subjects. 

 Plasma parameters regarding glucose and lipid homeostasis are used to differentiate the two obesity phenotypes^6,22,24^. As expected^11,22^, the MUO group, in the preoperative period, exhibited higher levels of blood glucose, insulin, HOMA-IR, TG, VLDL, GGT, ALT, ferritin, and urea than those observed for the MHO group, along with a reduction in HDL. After 1 year of bariatric operations, despite MUO subjects exhibiting glycemia, insulin sensitivity, lipid profiles, and urea plasma levels similar to those of MHO, they yet displayed AST, ALT, and ferritin plasma concentrations higher than MHO subjects, indicating that the bariatric procedure does not recover the low-grade inflammation and liver injury in MUO subjects. In addition, as observed by PCA, MUO subjects showed greater dispersion in their plasma metabolic and anthropometric characteristics, while MHO individuals exhibited less variation and more controlled changes after the bariatric operation, indicating that surgical interventions may have different effects on MHO and MUO individuals on such parameters. 

 Obesity progression is associated with ectopic fat deposition, especially in the liver, contributing to NAFLD development. This disease comprises one or various impairments in hepatic fatty acid metabolism, which lead to increased lipid deposition in hepatocytes, characterizing the first stage of the disease called simple hepatic steatosis. Such a condition can induce hepatic inflammation and fibrosis, progressing the disease to nonalcoholic steatohepatitis, which can progress to cirrhosis, and increase the risk of hepatocellular carcinoma^4,8,28^. Here, most of the MHO and MUO subjects in the preoperative period exhibited hepatic steatosis. After 1 year of bariatric surgery, hepatic steatosis was ameliorated in both obesity phenotypes, but approximately 30% of MUO subjects still exhibited hepatic steatosis. The literature lacks comparisons of the effects of bariatric surgery on hepatic steatosis according to different obesity phenotypes. Some early reports indicate that after 1 year of RYGB^25^ or SG^13^, some subjects still had hepatic steatosis and elevated plasma markers of liver injury. Our results suggest that most subjects exhibiting steatosis after bariatric surgery are from the MUO group. This indicates that this obesity phenotype may require a longer time to achieve disease remission and/or additional therapeutic interventions to improve liver health. 

 Finally, it is important to mention that despite the new data obtained in this study regarding bariatric effectiveness on MHO and MUO phenotypes, the study had several limitations. First, the sample size is small and predominantly of women, which prevented us from determining sex-specific differences. Second, as this was a retrospective study, we were limited to the biochemical data available in the medical records. Additional inflammatory markers, such as C-reactive protein, could have provided more comprehensive insights. Third, although the private service where the study was conducted performs a high number of bariatric surgeries, the most performed procedure was RYGB. Consequently, we were unable to determine which surgical type was more effective in improving the parameters analyzed in MHO and MUO subjects. Fourth, hepatic steatosis was assessed using ultrasound, which does not allow for the precise classification of NAFLD, since it is possible that MUO patients had non-alcoholic steatohepatitis rather than just hepatic steatosis. 

## CONCLUSIONS

 Bariatric surgery led to a similar effectiveness in weight loss, reductions in adiposity and anthropometric indices, and improvement in plasma glucose, lipids, insulin sensitivity, and renal parameters among MUO and MHO subjects. However, the effects of bariatric surgery between the MUO and MHO phenotypes differed significantly on hepatic steatosis and biochemical parameters of inflammation and liver damage, indicating that 1 year of the surgical intervention has better outcomes on hepatic steatosis in MHO subjects. 

## Data Availability

The Information regarding the investigation, methodology, and data analysis of the article is archived under the responsibility of the authors.
